# Gendered Dynamics of Contraceptive Decision-Making Among Currently Married Couples in Kerala, India: Insights From Qualitative Interviews

**DOI:** 10.7759/cureus.103796

**Published:** 2026-02-17

**Authors:** K Mukundhan Anusree, Sunu C Thomas

**Affiliations:** 1 Department of Public Health, Amrita Institute of Medical Sciences, Kochi, IND

**Keywords:** contraceptive choice, decision making process, gender equity, gender issues, gender norms, kerala, stigma

## Abstract

Introduction

Contraceptive use among couples is skewed towards limiting methods, with male method use remaining very low. This reflects a complex interplay of gender power relations in contraceptive decision-making. This study explored the perspectives and experiences of currently married women and men regarding contraceptive decision-making in Kerala, and aimed to understand how gender operates within these processes.

Methods

Qualitative in-depth interviews were done among currently married men and women in the age group 18-35 years in the Thiruvananthapuram and Thrissur districts of Kerala. An in-depth interview guide was used to capture the data. Data were collected from 19 participants until data saturation was obtained in the local language (Malayalam). Thematic analysis was done to generate the major themes.

Results

Findings indicate that gender power relations, masculinity norms, family and community expectations, and communication gaps between partners play a significant role in contraceptive decision-making among couples in Kerala. Women, due to a lack of knowledge, fear initiating conversation on contraception, while men assume it need not be talked about, as it is given that women have to undergo sterilization once the family size is complete. This indicated the deep-rooted gender norms that drive contraceptive decision-making. Men also do not approve of undergoing male sterilization, as it is stigmatized and threatens the masculinity norms in the community.

Conclusions

The study highlights the need to move beyond the assumptions of autonomous choice and recognize contraceptive decision-making as a negotiated process that is embedded in social relationships. Programs on family planning should aim to adopt gender-transformative strategies that engage men in the process. In addition, the programs should also aim to develop interventions that help couples to have open and gender-sensitive communication to help them make informed choices.

## Introduction

In India, contraceptive decision-making happens in a context shaped by patriarchal norms and power differences between partners. Despite policies emphasizing family planning and expanding contraceptive access, women’s agency in reproductive decision-making remains limited in many contexts, with gender dynamics exerting substantial influence on both the use and the type of contraception. Power relations within heterosexual unions are an important dimension of gender dynamics in contraceptive decision-making, and this is typically skewed towards men, resulting in unmet contraceptive needs when couples have to make contraceptive decisions. This imbalance is clearly noted in the methods mix pattern, with approximately 48% of currently married women reporting modern contraceptive use, and the majority of them relied on female sterilization (38%). While male sterilization accounted for less than 1% of the modern method uptake [1], the use of female-oriented methods not only reveals the gendered responsibility for fertility management but also signals how normative expectations about reproductive choices shape the method choice.

When egalitarian gender attitudes are absent or the husbands are not supportive of family planning, women resort to covert use of temporary methods. Literature shows that covert use is statistically associated with husbands’ less egalitarian gender attitudes, indicating that gender norms at the couple level can have a direct impact on joint decision-making [2]. Similarly, studies using couple dyad data show that male-female concordance on women’s involvement in contraceptive decision-making is linked to higher contraceptive use, while discordance between partners correlates with lower uptake. These indicate that when couples share mutual understanding about women’s agency, contraceptive utilization improves [3]. Gender dynamics in contraceptive decision-making in India are shaped by power asymmetries, normative expectations about gender roles, and a range of socio-cultural factors. Understanding these dynamics is important for designing family planning policies and interventions that not only increase access to contraceptives but also transform gender relations in order to achieve reproductive equity. Kerala achieved replacement-level fertility in the late 1980s, which is largely credited to the family planning initiatives and women’s literacy; however, the adoption and continuation of contraceptive methods are largely driven by partners’ decisions and family expectations [4]. Couples rely largely on female sterilization, which indicates that literacy and contraceptive access alone cannot tackle intra-household power asymmetries or equitable communication between couples around contraception [4,5]. Therefore, this study aims to explore the perspectives and experiences of currently married women and men regarding contraceptive decision-making in Kerala, and to understand how gender operates within these decision-making processes.

## Materials and methods

Study design

This study adopted a qualitative research design, and it was done among currently married men and women in the age group 18-35 years from two selected districts of Kerala. 

Study setting and participants

The study was conducted in the Thiruvananthapuram and Thrissur districts of Kerala. The districts were selected based on the overall contraceptive prevalence rate in Kerala. One with a high contraceptive prevalence (Thrissur-70%) and another with a low prevalence rate (Thiruvananthapuram-41%), compared to the state average (61%) [6]. Participants were selected from the community. 

Sample size and selection process

A total of 19 participants (six men and 13 women) were purposively selected. The number of participants was decided based on data saturation, i.e., when no new information was emerging from the interviews. Those who were currently married in the age group 18-35 years and those who were currently using or not using any methods of contraception were included in the study. Women who were pregnant at the time of data collection, as well as those whose husbands had not resided with them for the preceding three months, were excluded from the study. 

Participants were recruited using purposive sampling to ensure inclusion of currently married men and women in the age group 18-35 years. Initial contact was facilitated through Accredited Social Health Activists (ASHA) workers who informed eligible individuals about the study. Interested participants were approached directly by the first author, who provided detailed information about the study objectives and procedures.

Eligibility was assessed based on predefined inclusion and exclusion criteria, as specified, and willingness to participate. Individuals meeting the criteria and providing written informed consent were enrolled in the study. Recruitment continued until data saturation was achieved.

Data collection tools and techniques

Semi-structured, open-ended in-depth interview (IDI) guides were used to collect data from currently married women and men separately (Appendix). The IDIs were done by the lead author in the local language (Malayalam) after obtaining informed consent. The interviews were done face-to-face in the participant’s home. Each interview lasted 20-30 minutes. All interviews were audio-recorded with participants’ consent, transcribed verbatim, and translated into English for analysis. Data collection for the study was carried out from December 2024 to February 2025. 

Data analysis

Data were analyzed using Braun & Clarke's thematic analysis approach [7]. The interviews were analyzed using Python QualCoder version 2.2 (open-source software).

The first author did the open coding. The analytic process began with repeated reading of each transcript to achieve familiarization with the data. An inductive approach was adopted, allowing patterns and meanings to emerge from participants' narratives. Transcripts were then coded line by line, with codes assigned to each meaning unit of text. Following initial coding across all transcripts, codes were reviewed by both authors and compared to identify patterns of shared meaning. Related codes were then collated and formed the categories. 

Themes were developed subsequently through iterative comparison between coded extracts and the full dataset to ensure internal coherence within themes and a clear distinction between themes. Illustrative participant quotations were selected to demonstrate how interpretations were grounded in participants’ accounts. The second author also engaged in the iterative process to refine the themes. These steps were repeated for transcripts of men and women separately, and were subsequently looked for any convergence and divergence across groups. Table 1 shows an illustrative example of the coding process. 

**Table 1:** Illustrative coding process.

Verbatim quote	Open codes	Category	Theme
“My husband doesn’t prefer condoms… I prefer condoms. But since he is not interested, I am forced to use other methods.” (Female Participant 6)	Women forced to used methods they are not interested	Limited negotiations in contraceptive decision-making	Gendered power relations in contraceptive decision-making
“Usually, the husband takes the final decision. We can only suggest.” (Female Participant 3)	Husband as the final authority in contraceptive decision-making		
“Even if I have side effects, I adjust.” (Female Participant 9)	Endurance of side effects	Gendered normalization of female responsibility	
“Beginning of marriage, it is difficult to talk about these things.” (Female Participant 1)	Communication difficulties early on in marriage	Communication barriers	Communication barriers due to gendered expectations
“We feel shy to bring it up.” (Female Participant 5)	Feeling of shyness to talk discuss contraceptives		
“It is very shameful for a woman to go and get that.” (Female Participant 12)	Fear of judgment and stigma	Gendered moral surveillance	
“Yes, men can do vasectomy. But they are afraid.” (Female Participant 6)	Fear of vasectomy	Resistance to male sterilization	Masculinity and fear around male contraception
“If a man does operation, people will talk.” (Female Participant 10)	Social ridicule of men undergoing vasectomy	Masculinity under public gaze	
“My family insisted my sister to put CuT… she suffered.” (Female Participant 4)	Family pressure to use certain contraceptives	Family-mediated decision-making	Family and community expectations
“Yes. It is difficult for women to access contraceptives. You know our society will always judge, especially when women go out for these things. They will say that women are in another relationship like that. But if the husband is supportive, nothing else matters. We can’t shut their mouth.” (Female Participant 9)	Community monitoring/judgement	Community surveillance of reproduction	
“If she wants, we will discuss. But finally, I decide what is practical.” (Male Participant 15)	Conditional consultation with the female partner	Male control framed as practicality	Gendered power relations in contraceptive decision-making
“Sometimes women don’t understand these things properly.” (Male Participant 16)	Dismissal of women’s decision-making capacity	Knowledge as a source of male control	
“We don’t openly talk. These things are understood.” (Male Participant 17)	Avoidance of discussion on contraception due to the belief that it's implicitly understood	Normalization of silence on contraceptive decision-making	Communication barriers due to gendered expectations
“We won’t discuss much. You know, after having kids we don’t have time for that. Even for basic things we won’t be getting time. So, about these things, who cares? And also, now that she has stopped the pregnancy, there is no need of discussing.” (Male Participant 17)	Normalization of not communicating about contraception after children		
“Men can do vasectomy. But they are afraid… These men won’t do that.” (Female Participant 6)	Fear of vasectomy	Resistance to male sterilization	Masculinity and fear around male contraception
“After vasectomy, people will say the man has become weak.” (Male Participant 18)	Masculinity linked to strength		
“Condom reduces pleasure.” (Male Participant 14)	Method rejection due to sexual dissatisfaction	Male-centered contraceptive evaluation	
“But since I have only one female child, even my relatives used to say we should have one male child also to keep the generation forward. So, there will be pressure from the family to have a male child. Now things have changed. But still, it’s there. If I have one male child and if we go for sterilization, people won’t be saying anything, but if I only have one girl child and do sterilization, people will even consider me as a fool.” (Male Participant 15)	Familial pressure to continue the male lineage	Reproductive decision-making is shaped by extended family/gendered expectations	Family and community expectations

Ethical considerations

The study was cleared by the Ethics Committee of Amrita Institute of Medical Sciences with the approval ID: ECASM-AIMS-2024-601 dated 13-12-2024. Written informed consent was obtained from all the participants prior to the interview. Consent was obtained to audio-record the interview. The privacy of participants was maintained throughout the interview. All identifying information was anonymized while presenting the findings of the study.

## Results

Table 2 shows the participant characteristics. The findings of the study are summarized under four themes: gendered power relations in contraceptive decision-making, communication barriers due to gendered expectations, masculinity and fear around male contraception, and family and community expectations.

**Table 2:** Characteristics of the participants.

Participants	Sex	Age	Education	Occupation	Current contraceptive method	Contraceptive decision
Participant 1	Female	30	Higher	Not employed	Condoms	Self
Participant 2	Female	35	Up to secondary	Employed	Copper T	Male partner
Participant 3	Female	26	Higher	Not employed	No methods	Male partner
Participant 4	Female	29	Higher	Not employed	No methods	Jointly
Participant 5	Female	25	Higher	Employed	Copper T	Self
Participant 6	Female	32	Higher	Employed	No methods	Jointly
Participant 7	Female	21	Higher	Not employed	No methods	Self
Participant 8	Female	29	Higher	Not employed	No methods	Jointly
Participant 9	Female	26	Higher	Employed	Condoms	Jointly
Participant 10	Female	31	Higher	Not employed	No methods	Jointly
Participant 11	Female	32	Higher	Employed	I pill	Male partner
Participant 12	Female	35	Higher	Employed	Condoms	Jointly
Participant 13	Female	30	Higher	Not employed	Natural methods	Jointly
Participant 14	Male	32	Higher	Employed	Condoms	Jointly
Participant 15	Male	35	Higher	Employed	Condoms	Jointly
Participant 16	Male	35	Up to secondary	Employed	Tubectomy	Jointly
Participant 17	Male	33	Up to secondary	Employed	No methods	Self
Participant 18	Male	34	Higher	Employed	Tubectomy	Jointly
Participant 19	Male	35	Higher	Employed	Tubectomy	Jointly

Gendered power relations in contraceptive decision-making

Gendered power dynamics operate even before the actual decisions are made in contraceptive decision-making among couples in Kerala. Women fear initiating any conversation with their partners, as they have a perceived lack of knowledge and misconceptions about contraceptives. They also felt their partners were completely unaware of the contraceptive methods, either, which also hindered them from initiating any conversations on it. Some women said they secretly use some method since they fear opposition if they were to discuss it. Men think it’s the responsibility of women to think of contraception and mentioned that it’s an unwritten rule that women will do sterilization after the second or third pregnancy. Hence, according to men, there was no need for any discussion on it. 

“Women are more responsible than men because it is related to their body. But men also should support her.” - Male Participant 15

“There is no decision like that. It is a practice; all women after 2 or 3 pregnancies will do sterilization*.*” - Male Participant 16

“Then within one year after my third delivery, I got pregnant again. But that led to miscarriage. Again, in six months another miscarriage happened. That time I got admitted to the hospital for a few days. My husband is eight years older than me; he is very loving and caring, but he cannot understand these things. Doctors also scolded me. Hence, after I got discharged from the hospital, I was staying with my family. That time I went and put in a copper T. My husband doesn’t know about it still*.*” - Female Participant 2

Communication barriers due to gendered expectations

Limited communication regarding contraceptive decision-making is influenced not only by a lack of knowledge but also by the gendered expectations about the roles that they have to play within marriage. Men avoided the intimate conversations around contraception due to norms around emotional expression, while women avoided these due to fear of conflict and stigma. It was also shaped by parenting responsibilities and workload, which left no time to discuss contraception. Also, it was not given any priority within the marriage. 

“We won’t discuss much. You know, after having kids we don’t have time for that. Even for basic things we won’t be getting time. So, about these things, who cares? And also, now that she has stopped the pregnancy, there is no need of discussing*.*” - Male Participant 17

“Not much… He is also not having much knowledge about it (contraceptives), I think. Then when we have other problems in our life, we won’t have time to think about this*.*” - Female Participant 10

Masculinity and fear around male contraception

Men's engagement with contraceptive use was influenced by norms of masculinity and fear of stigma. Vasectomy as a method of contraception was often seen as unacceptable by men, who expressed it as “uncommon” in society and expressed discomfort. Women also reported stigma when men adopted contraception. 

“Firstly, I don’t know anything about this (vasectomy). Like I don’t know anyone who did that also either. It’s not a common practice in our society. That’s all I know…” - Male Participant 16

“Yes, there is a stigma in the society when males do sterilization. But people and their attitudes are changing. Many of us don’t know about contraception-related things.” - Male Participant 19

“There is a lot of stigma around men opting for sterilization.” - Female Participant 13

Family and community expectations

Decisions to use contraception were made based on socio-cultural norms, family pressure, and community expectations. The preference for a male child played an important role in this. In addition to these, religious beliefs and stigma around women accessing contraceptives further exacerbated the power imbalances in decision-making. 

“Yes. It is difficult for women to access contraceptives. You know our society will always judge, especially when women go out for these things. They will say that women are in another relationship like that. But if the husband is supportive, nothing else matters. We can’t shut their mouth.” - Female Participant 9

“But since I have only one female child, even my relatives used to say we should have one male child also to keep the generation forward. So, there will be pressure from the family to have a male child. Now things have changed. But still, it’s there. If I have one male child and if we go for sterilization, people won’t be saying anything, but if I only have one girl child and do sterilization, people will even consider me as a fool*.*” - Male Participant 15

## Discussion

Our study shows that contraceptive decisions are negotiated within unequal power structures. Similar findings are reported in other studies, which show that husbands hold power within marital relationships, even when reproductive health decisions need to involve both partners. While family planning decisions have to be shared, they predominantly occur in contexts where men have more authority and privilege [8,9]. 

Women reported limited autonomy and often complied with their partners' preferences or engaged in covert use. There is evidence that women circumvent male authority by covert use of contraceptive [10] to divert blame. Lack of communication regarding contraception was noted in the couples in the study, which is consistent with findings showing that couple communication predicts contraceptive use, with equitable communication resulting in greater use [11]. Women adjusting silently to male preferences or not being able to negotiate what they prefer clearly indicates gendered inequalities in contraceptive decision-making. Men’s view that contraception is the responsibility of women also reinforces the gender norms and roles within the society [12]. Couple communication can play a crucial role in making informed decisions in reproductive health, particularly in contexts where social and gender norms force couples to conform [11]. Literature shows an association between spousal communication and adoption of modern contraceptives [13,14]. This becomes even more important considering the attitude of men in the study. 

Men in our study were not proactive in contraceptive decision-making and also expressed discomfort with the use of male methods like vasectomy. This reflects the masculine norms that discourage active engagement in reproductive health. Research shows that even when men endorse shared responsibility for contraception, they do not take part in active decision-making, placing the burden of preventing pregnancy on women. This dynamic can limit women’s ability to negotiate their own preferences and adjust to male expectations [15]. Findings from NFHS-4 data showed that men’s attitudinal norms were negatively associated with contraceptive use by women [16]. These patterns illustrate that the preference for or use of certain methods and avoidance of male methods are not only about knowledge or misconceptions but also about masculinity and control over reproductive agency, which in turn can negatively affect women’s health. Considering the high literacy rates (96.1% for males and 92.1% for females) in Kerala [17], the findings of a lack of awareness and male attitudes towards contraception have to be seriously considered. 

Our findings also show that the decisions regarding contraception were influenced by family and societal expectations, particularly of male child preference. There is also a socio-cultural stigma associated with women’s access to contraceptives. Evidence suggests that cultural and gender dynamics restrict women’s autonomy in family planning [18]. Studies have mentioned that women's empowerment is associated with modern contraceptive use. While this is true, the finding from our study throws light on the gendered aspects of the decision-making process [19,20]. The findings of the study highlight the importance of addressing gendered communication dynamics between partners rather than solely focusing on women's self-reported autonomy [3]. Women's empowerment may be beneficial, but addressing the root causes, such as harmful social and gender norms, may offer long-term benefits to women and children. Evidence from a systematic review shows that prevailing gender norms in a community can restrict women’s autonomy in reproductive health and family planning, reiterating that these norms act as a hindrance to women’s sexual and reproductive health outcomes and underscore the need for interventions targeting harmful social and gender norms for long-term benefits [18]. 

This study has several strengths. The use of in-depth qualitative interviews enabled rich exploration of the relational and gendered processes underlying contraceptive decision-making. Interviewing men and women separately allowed for discussions of sensitive issues related to power, masculinity, and negotiation within marriage. Conducting interviews in participants’ homes and in the local language enhanced contextual sensitivity and cultural validity. The use of reflexive thematic analysis facilitated an in-depth, process-oriented understanding of shared patterns across narratives.

However, there were certain limitations to the study. The study was conducted within a specific socio-cultural context in Kerala, which may limit transferability to other settings. The absence of dyadic interviews restricted direct observation of couple-level negotiation processes. Given the sensitive nature of discussions surrounding contraception and marital dynamics, responses may have been influenced by social desirability bias. Although interviews were conducted privately and confidentiality was emphasized, participants may have presented their experiences in ways that align with socially accepted norms. Additionally, while a semi-structured guide was used to ensure consistency, the flexible nature of qualitative interviewing may have led to variation in probing across interviews.

## Conclusions

Our findings indicate that contraceptive decision-making cannot be understood in isolation from gender norms and relational power structures. Therefore, interventions should adopt gender-transformative strategies that engage men in challenging normative ideas of masculinity and reproductive responsibility. It should also aim for open, gender-sensitive communication between couples as well as address community and family-level stigma that constraints contraceptive choices. Such approaches should move beyond awareness campaigns and target deep-rooted gendered dynamics that shape reproductive health outcomes. While the findings are context-specific and not generalizable, they offer transferable insights into how gendered relationships influence reproductive health behaviors.

## Appendices

In depth interview guide

Female Participants

1. Have you heard about family planning? If yes, can you elaborate on it (probe: what is it. Where have you heard about it)

2. Do you know about temporary and permanent methods of contraception? (probe: which method is more familiar to you? From where have you heard about these methods?)

3. What kind of methods are generally used by women? (probe: Why do you think they use that method? What are your thoughts on permanent methods like tubal ligation?)

4. What is the current method that you are using? can you explain why you choose to use this method? How did you come to decide on this particular method - in discussing with the partner, family member or friends?

5. Do you believe women have a role in deciding the type of contraceptive method to be used? Why do you say so?

6. What is your role in deciding contraceptives in your marriage?

7. How do you communicate to your partner regarding the contraceptive methods that you use? (probe: in the beginning of marriage, later, after one child/children. Who initiated the conversation)

8. Can you explain whether family expectations have a role in contraceptive decision making and how? How does your family intervene in your contraceptive decision making?

9. Do cultural beliefs have a role in influencing contraceptive decision making? If so, can you elaborate on it

10. Do you think your religious beliefs plays a role in contraceptive decision making?

Male Participants

1. Have you heard about family planning? If yes, can you elaborate on it (probe: what is it. Where have you heard about it)

2. What is the current method that you are using? Can you explain why you choose to use this method?

3. In your opinion, who is more responsible for adopting a contraceptive method? Do you think family planning is a women’s responsibility? (if yes explain why?)

4. Would you consider adopting vasectomy as a method of sterilization, and what factors would influence your decision?

5. Can you explain whether family expectations have a role in contraceptive decision making and how? How does your family intervenes in your contraceptive decision making?

6. Do cultural beliefs have a role in influencing contraceptive decision making? If so, can you elaborate on it

7. Do you think your religious beliefs plays a role in contraceptive decision making?
